# Modeling the precise interaction of glioblastoma with human brain region-specific organoids

**DOI:** 10.1016/j.isci.2024.109111

**Published:** 2024-02-05

**Authors:** Qi Fan, Hanze Wang, Tianyi Gu, Huihui Liu, Peng Deng, Bo Li, Hui Yang, Ying Mao, Zhicheng Shao

**Affiliations:** 1 Institutes for Translational Brain Research, State Key Laboratory of Medical Neurobiology, MOE Frontiers Center for Brain Science, Institute of Pediatrics, National Children’s Medical Center, Children’s Hospital, Fudan University, Shanghai, China; 2 Department of Neurosurgery, Huashan Hospital, Institute for Translational Brain Research, State Key Laboratory of Medical Neurobiology, MOE Frontiers Center for Brain Science, Fudan University, Shanghai 200032, China; 3 Human Phenome Institute, Zhangjiang Fudan International Innovation Center, Fudan University, Shanghai 201203, China; 4 National Center for Neurological Disorders, Huashan Hospital, Fudan University, Shanghai 200040, China; 5 Shanghai Key Laboratory of Brain Function Restoration and Neural Regeneration, Huashan Hospital, Fudan University, Shanghai 200040, China

**Keywords:** Microenvironment, Molecular biology, Neuroscience, Techniques in neuroscience, Omics, Transcriptomics

## Abstract

Glioblastoma is a highly aggressive malignant tumor of the central nervous system, but the interaction between glioblastoma and different types of neurons remains unclear. Here, we established a co-culture model *in vitro* using 3D printed molds with microchannels, in which glioblastoma organoids (GB), dorsal forebrain organoids (DO, mainly composed of excitatory neurons), and ventral forebrain organoids (VO, mainly composed of inhibitory neurons) were assembled. Our results indicate that DO has a greater impact on altered gene expression profiles of GB, resulting in increased invasive potential. GB cells preferentially invaded DO along axons, whereas this phenomenon was not observed in VO. Furthermore, GB cells selectively inhibited neurite outgrowth in DOs and reduced the expression of the vesicular GABA transporter (VGAT), leading to neuronal hyperexcitability. By revealing how glioblastoma interacts with brain cells, our study provides a more comprehensive understanding of this disease.

## Highlights

• Establishing a 3D model for exploring glioblastoma-neural organoid interactions
• Glioblastoma cells preferentially invade dorsal forebrain organoids
• Glioblastoma cells selectively inhibit axonal growth in dorsal forebrain organoids
• Glioblastoma cells increase neural activity in dorsal forebrain organoids

## Graphical abstract

**Figure:**
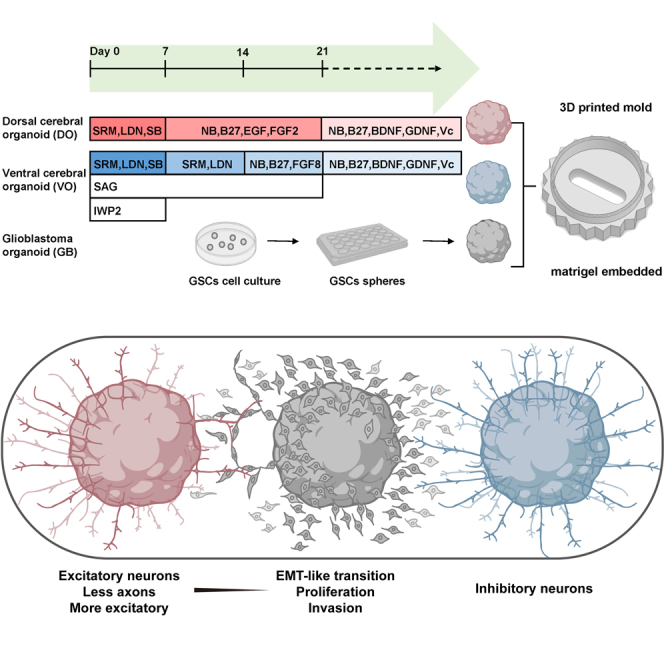

## Introduction

Glioblastoma is an aggressive, highly malignant central nervous system tumor characterized by aggressive growth, widespread dissemination, and rapid progression.^1^ A study of 331 cases reported that 40% of cases were in the frontal lobe, 29% in the temporal lobe, 14% in the parietal lobe, 3% in the occipital lobe, and 14% in deep structures.^2^ Such regional differences may be attributed to differences in neuronal cell types and population dynamics within different brain regions.^3^ However, the exact underlying factors that contribute to susceptibility to glioblastoma in the human brain remain elusive, highlighting the need for further research.

Growing evidence demonstrates that glioma cells can directly or indirectly interact with neurons.^4^ For example, glioblastoma cells can establish connections with brain cells through microtubules, and the formation of neurogliomal synapses has been observed in invasive tumor areas.^5^ These adjacent brain cells form a unique tumor microenvironment that plays a critical role in glioma progression. The paracrine effect between glioma and brain cells is specific to the cell types involved.^6^ Glutamate stimulates the growth of glioma,^7^ while gamma-aminobutyric acid (GABA) signals continuously inhibit it.^8^ Without GABA signals, the disease progression accelerates. However, most previous studies have relied on two-dimensional cell cultures or mouse patient-derived xenografts (PDX) models, lacking a human tumor microenvironment.^9^ In recent years, organoids have emerged as a valuable tool for studying various cancer models, including brain organoids that enable the investigation of tumor-neuron interactions.^10^^,^^11^^,^^12^^,^^13^ Nevertheless, the specificity interaction between glioma and different types of human neurons remains largely unexplored.

In this study, we developed a method to co-culture glioblastoma organoids with region-specific brain organoids using 3D printed molds, which can be used to model the *in vivo* mechanisms of tumor cell-neuron interactions. Specifically, we induced dorsal forebrain organoids (DO), which are mainly composed of excitatory neurons, and ventral forebrain organoids (VO), which are mainly composed of inhibitory neurons. Combined with glioblastoma organoids (GB) derived from glioma stem cells 3264, we used Matrigel to establish three co-culture models, as follows: (1) DOs and VOs positioned on the left and right sides of GB, (2) two DOs located on the right side of GB, one in proximity and the other further away, and (3) two VOs situated on the right side of GB. Compared to VOs, DOs preferred to alter the profile of gene expression of GB cells, which demonstrated a highly invasion potential. We found that GBs were prone to invade DOs, selectively inhibited the neurite growth, and decreased the expression of VGAT, which may finally lead to neuronal hyper-excitability. In summary, our study sheds light on the complex interactions between glioblastoma and different types of neurons. By establishing a co-culture model that replicates the *in vivo* microenvironment, we provide valuable insights into the biological characteristics of glioblastoma and its impact on brain cells. These findings may inspire the development of precise treatment strategies targeting distinct brain regions affected by glioblastoma.

## Results

### Induction of dorsal/ventral forebrain organoids and glioblastoma organoids

**Figure 1:**
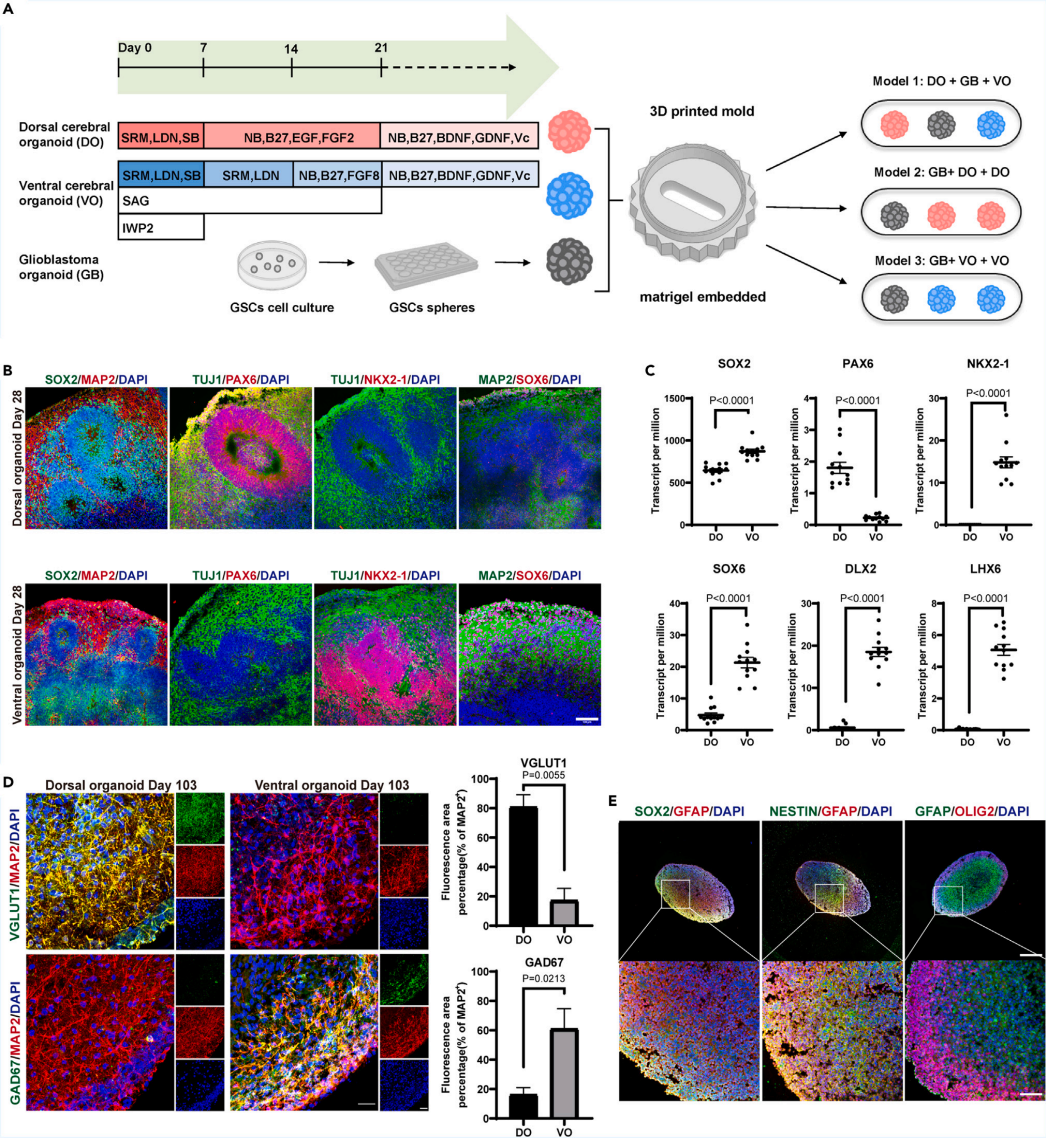
Establishment a new co-culture model of dorsal/ventral forebrain organoids and glioblastoma organoids (A) Schematic of organoids protocol and co-culture model. SRM, serum replacement medium; LDN, 100 nM LDN193189; SB, 10 μM SB431542; SAG,0.1 μM Smoothened agonist; IWP2, 5 μM inhibitor of Wnt production 2; NB, neural basal medium. (B) Images of dorsal and ventral forebrain organoids immunostained for SOX2, PAX6, NKX2-1 and SOX6. Scale bars, 100 μm. (C) Transcripts per million (TPM) of the expression of indicated markers in dorsal and ventral forebrain organoids. Values represent mean ± SEM (n = 12). (D) Images of dorsal and ventral forebrain organoids immunostained for VGlut1 and GAD67. Scale bars, 50 μm. Percentage statistics of fluorescence intensity area. Values represent mean ± SEM (n = 3). (E) Images of GBM organoids immunostained for SOX2, GFAP, NESTIN and OLIG2. Scale bars, 500 μm and 100 μm.

Dorsal/ventral forebrain organoids (DOs/VOs) were generated following a previously established protocol with slight modifications (Figure 1A).^14^^,^^15^ Immunostaining analysis revealed the expression of SOX2, a marker for neural progenitor cells, in the ventricular zone (VZ) of both dorsal forebrain organoids (DOs) and ventral forebrain organoids (VOs) on day 28. MAP2^+^ neurons were observed outside the VZ. The cells in VZ regions of DOs primarily expressed the dorsal forebrain marker PAX6+, while lacking the ventral forebrain marker NKX2.1. In contrast, VOs exhibited abundant NKX2.1+ progenitor cells with only a few PAX6+ cells. Additionally, SOX6, which regulates forebrain progenitor and interneuron heterogeneity, showed low expression levels in DOs but high expression in VOs (Figure 1B). RNA-sequencing analysis of 6-week-old DOs and VOs confirmed the consistent expression patterns of SOX2, PAX6, NKX2.1, and SOX6 observed in the immunostaining results (Figure 1C). Furthermore, we investigated the specific markers within distinct sub-regions of the ventral forebrain ganglionic eminence, known for generating diverse subtypes of interneurons. Notably, DLX2, a marker for ventral forebrain development, showed higher expression in VOs than in DOs, and there was also a significant increase in LHX6 expression (Figure 1C), confirming the successful establishment of ventral identities in VOs. We observed that the persistence of the VZ formed by SOX2^+^ cells still existed in DO at 60 days (Figure S1A). Furthermore, the expression of excitatory neuronal markers spanning the six cortical layers was detected in DOs. We found that TBR1^+^ deep-layer neurons along with a few CTIP2^+^ neurons were expressed in the subventricular zone (SVZ), representing immature neurons in this region (Figures S1B and S1C). Upper-layer cortical neurons positive for SATB2 and Reelin were located near the pial surface (Figures S1A and S1C), resembling the marginal zone (MZ) layer I of the human cortex *in vivo*. These results revealed that our induced DOs generated distinct neuronal subtypes across the six cortical layers, exhibiting a distribution similar to the developing human cortex (Figure S1D). By day 105, the average population of VGlut1^+^ excitatory neurons was 80.87% in DOs and only 17.22% in VOs (Figure 1D). In contrast, GAD67^+^ inhibitory neurons accounted for 16.15% in DOs and a significantly higher proportion of 61.05% in VOs. (Figure 1D). Together, these results confirmed that our induced dorsal forebrain organoids (DOs) primarily consisted of excitatory neurons, whereas ventral forebrain organoids (VOs) predominantly comprised inhibitory neurons, providing a suitable model for investigating the interaction between these distinct neuronal populations with glioblastoma.

We generated glioblastoma organoids (GBs) using glioblastoma stem cell line 3264 and assessed the growth rate (Figures S1E and S1F). To further characterize the cellular properties of GB, we performed immunostaining analysis using the markers including GFAP, OLIG2, NESTIN, and SOX2 (Figure 1E). *In vivo*, glioma stem cells (GSCs) with tumor self-renewal ability and aggressive growth metastasis are typically found in the perivascular niche,^16^ expressing SOX2, OLIG2, and NESTIN. On the other hand, GFAP indicates the differentiation of GSCs. Our results revealed regional heterogeneity in the tumor-like structures of GBs, with OLIG2^+^ stem cell populations occupying hyperoxic, highly nutritious regions at the periphery, while GFAP^+^ differentiated cells resided in chronic hypoxic necrotic regions at the core. In summary, our induced glioblastoma organoids showed the in vivo-like features of glioblastoma.

### Establishment of the new co-culture models using 3D printing

To study the interaction between excitatory and inhibitory neurons and glioblastoma, we used 3D printing technology to create a special culture dish with channel dimensions of 7 mm wide, 24 mm long, and 5 mm high, which was designed to provide an optimal growth environment for co-culturing 3D organoids (Figures S1G and S1H). The organoids were placed at appropriate distances within the channel, ensuring their spatial alignment, and then embedded in Matrigel, which mimics the extracellular matrix *in vivo*. This embedding process not only promoted cell migration and growth, but also allowed the direct visualization of GB invasion processes and the axonal growth of DO and VO. Moreover, it improved the separation of different organoid types during subsequent experimental procedures.

To study the direct and indirect interactions between DO, VO, and GB, we established three distinct co-culture models (Figure 1A): (1) GB positioned in the middle of DO and VO; (2) DOs proximal/distal from GB; (3) VOs proximal/distal from GB. The first model enabled a direct comparison of the interactions between DO and GB, as well as between VO and GB. We assigned a unique name to each organoid in the first model as GB.1, DO.1, and VO.1 respectively. In the second or third models, we independently examined the direct and indirect interactions by varying the distance between DO and GB or VO and GB. In the second model, we labeled three types of organoids as GB.2, DO.2n (near), and DO.2f (far). Similarly, in the third model, organoids were labeled as GB.3, VO.3n (near), and VO.3f (far). As control groups, GBs, DOs, and VOs were individually embedded in Matrigel at the same time, referred to as ctrl GB, ctrl DO, and ctrl VO, respectively.

### Glioblastoma cells preferentially invaded dorsal forebrain organoids

**Figure 2:**
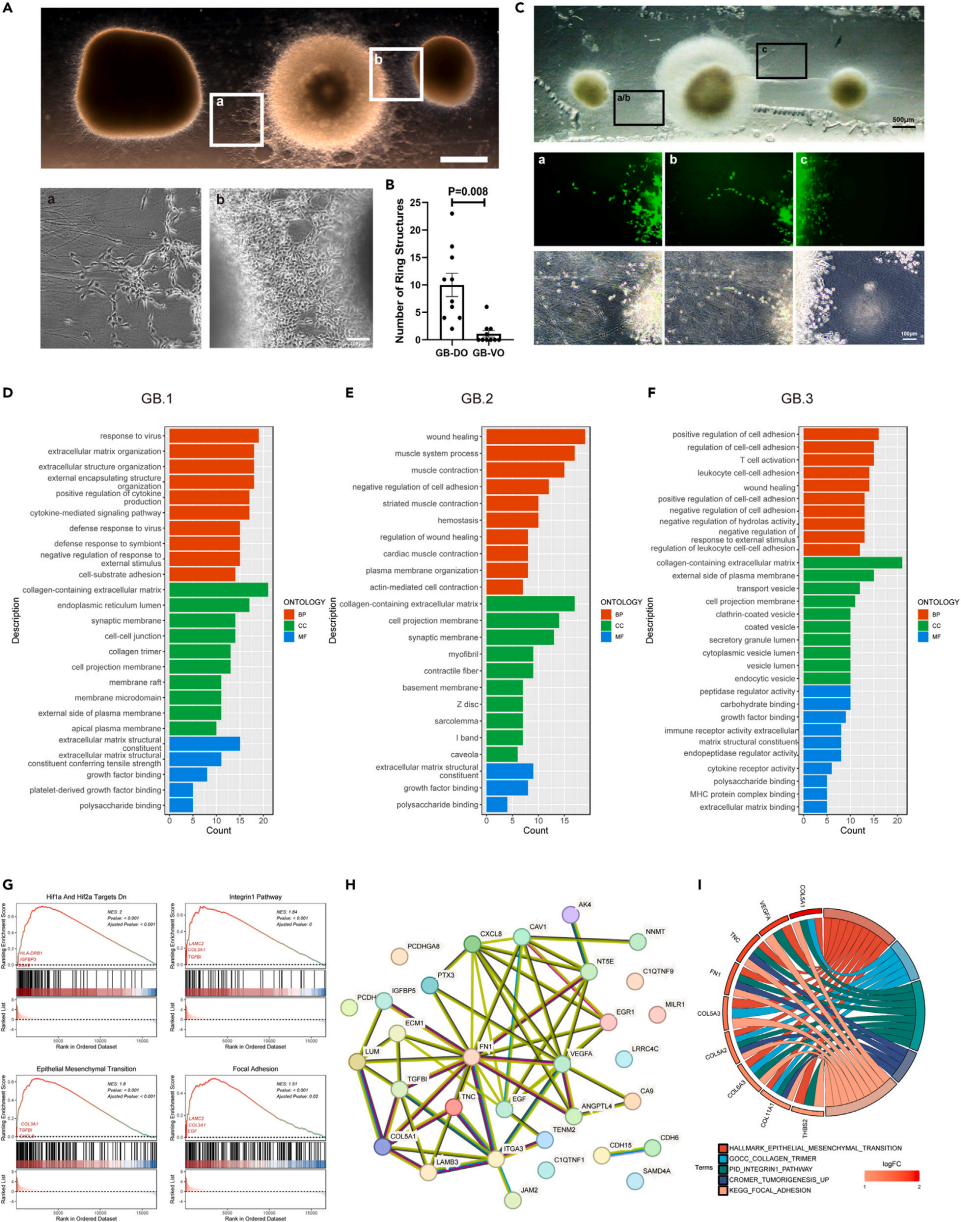
Glioblastoma cells preferentially invaded dorsal forebrain organoids (A) Morphology of GB cells under stereopicroscope and phase contrast microscope. From left to right, DO, GB, VO. Scale bars,1000 μm and 100 μm. (B) Statistics of the number of ring structures between DO-GB and VO-GB. Values represent mean ± SEM (n = 10). (C) Images of invasive GB cell populations at the outer edge of the GB. Scale bars, 500 μm and 100 μm. (D–F) Gene Ontology terms in GB.1, GB.2 and GB.3. (G) GSEA plot of GB.2 vs. ctrl.GB. (H) Protein network interaction diagram of related DEGs in GB.2. (I) Chord diagram showing key DEGs in GB.2 involved in related pathways.

In our study, we observed the proliferation and migration of glioblastoma cells in Matrigel, leading to invasion and metastasis toward DOs or VOs. In the first model, GB displayed elongated morphological changes and exhibited a preference for invading to DOs (Figure 2A). These morphology changes may be associated with cytoskeletal changes and the loss of cell polarity, indicating the presence of a highly invasive mesenchymal subtype of GB.^17^^,^^18^ Additionally, transformed GB cells were observed to form a ring-like structure by connecting with each other. We observed a significant increase in ring-like structures in the GB-DO co-culture compared to the GB-VO co-culture, indicating a higher number of cells undergoing morphological transformation in the GB-DO co-culture (Figure 2B). Furthermore, we observed the migration of GB cells along the axons of DOs in Matrigel (Figure 2A), while this phenomenon was not observed in VOs. To visualize the invasion activities of GB cells more precisely, we transduced glioma stem cells with a GFP lentivirus and then co-cultured with DOs and VOs. After 7 days of co-culture, the axons of DOs and VOs extended to the surface of GB. We detected GFP^+^ GB cells migrating along axons of DO and undergoing further division and proliferation within 24 h (Figures 2C-a, b). However, despite the presence of numerous axons from the VOs in contact with GB, the proliferated GB cells did not migrate along the axons of the VOs (Figures 1C-c). Taken together, these results suggest that GB cells show a preference for invading DOs rather than VOs in our *in vitro* cultured system.

### Enhancing invasive gene expression of glioblastoma organoids cells by interacting with neurons

To explore the molecular mechanism underlying the observed phenomena, we conducted RNA sequencing analysis of the organoids from three co-culture models and the control organoids after 14 days of co-culture. Principal component analysis (PCA) demonstrated clear clustering between the co-cultured organoids and the control groups, indicating substantial transcriptional changes (Figure S2A). Differentially expressed genes (DEGs) analysis identified a set of DEGs, as evidenced by the Venn and volcano diagrams (Figures S2B–S2F). Notably, there was a significant enrichment of upregulated genes compared to downregulated genes in all the three GBs models (Figures S2B and S2C), with GB.2 exhibiting a larger number of DEGs than GB.3 (Figures S2B and S2C). Gene Ontology (GO) enrichment analysis of the DEGs in GB.2 revealed a significant enrichment in biological processes related to the negative regulation of cell adhesion and actin-mediated cell contraction (Figure 2E). These DEGs were also associated with various cellular components, including collagen-containing extracellular matrix, cell membrane projections, myogenic and contractile fibers, as well as molecular functions associated with growth factor binding and polysaccharide binding. Notably, these upregulated pathways align with previous studies on DEGs between low- and high-risk groups of patients with glioblastoma under epithelial-mesenchymal transition (EMT)-related long non-coding RNA labeling.^19^ In addition, GB.1 exhibited enrichment of DEGs related to extracellular matrix structure, while DEGs in GB.3 were predominantly associated with immune system activity, encompassing T cell activation, immune receptor activity, and MHC protein complex binding (Figure 2F). Meanwhile, DEGs between GB.2 and GB.3 were also found on cell adhesion and immune-related pathways (Figures S3A and S3B).

Furthermore, Gene Set Enrichment Analysis (GSEA) using the MSigDB database was performed on GB.2 and ctrl.GB, revealing the activation of the HIF response system and integrin 1 signaling pathway in GB.2. Additionally, we observed elevated expression levels of genes associated with epithelial-mesenchymal transition (EMT) and focal adhesion, both processes tightly linked to tumor invasion (Figure 2G). We further investigated the expression patterns of CDH1 (Cadherin1), CDH2, CDH11, and VIM (Vimentin), which are involved in cell adhesion and migration.^20^^,^^21^^,^^22^ Remarkably, GB cells from all groups exhibited low expression of CDH1, a downward trend in CDH2 expression, and significantly upregulated expression levels of CDH11 and VIM (Figure S3C). These findings suggested that the neural activity of DOs compared with VOs may affect the migration and invasive potential of GB cells, similar to the process of mesenchymal transition, in which cells acquire a more aggressive phenotype in response to environmental cues. Moreover, excitatory neural activity may be more prominent in enhancing this invasive behavior.

In addition, we also identified several key genes involved in tumor aggressiveness, including COL5A1, VEGFA, TNC, and FN1 (Figures 2H and 2I). TNC, which is upregulated in mesenchymal glioblastoma subtypes with high NF-κB signaling activity, has been shown to promote glioma stem cell proliferation.^23^ Additionally, fibronectin FN1 plays an essential role in promoting GBM aggressiveness through GBP2.^24^ In our co-culture model, these genes exhibited significant upregulation (Figure 2I) and were consistent with the expression levels observed in patient tumors (Figure S3D). Importantly, their elevated expression was more pronounced in GB than in low-grade gliomas and correlated with poorer survival outcomes (Figure S3E). Collectively, our co-culture models effectively mimicked the invasive processes of GB at both cellular phenotype and gene expression profiles, demonstrating their promising potential for investigating the tumor microenvironment.

### Glioblastoma cells selectively inhibited axonal growth and increased neural activity in dorsal forebrain organoids

**Figure 3:**
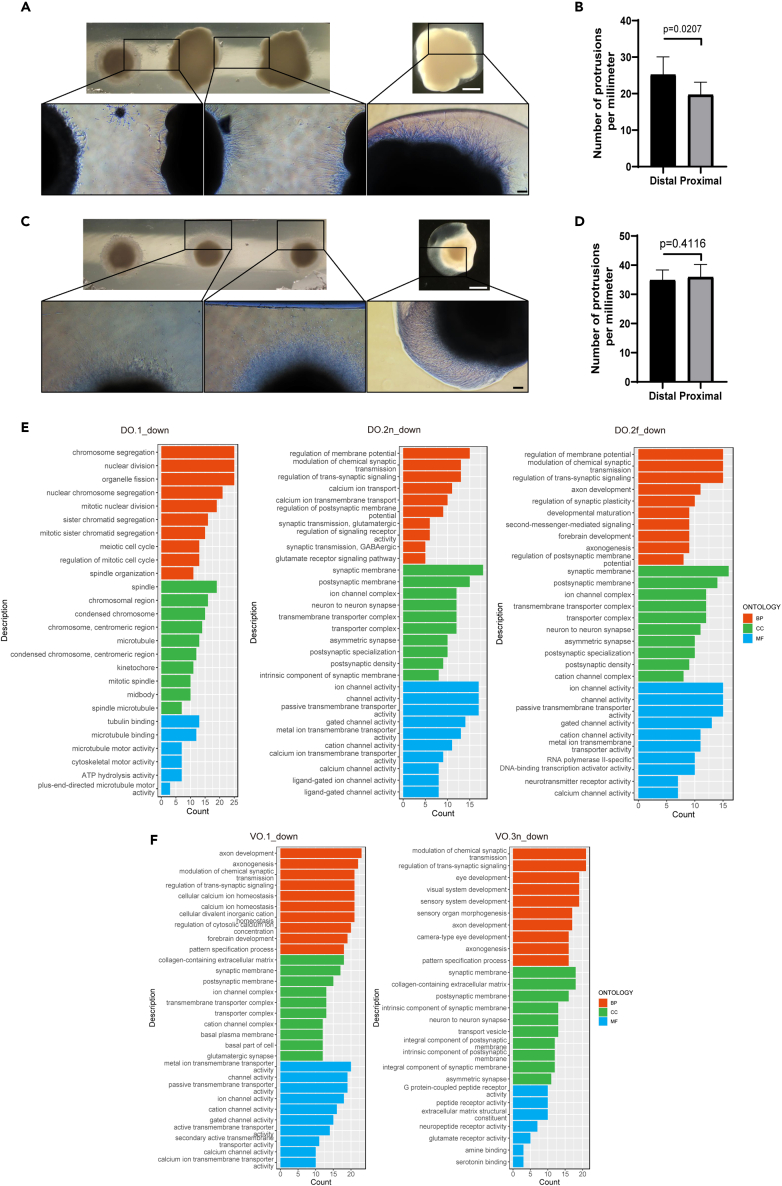
Glioblastoma cells selectively inhibited axonal growth in dorsal forebrain organoids (A and C) Axon growth of DOs and VOs after co-culture under stereopsis and phase contrast microscopy. From left to right are GB.2, DO.2n, DO.2f (A) and GB.3, VO.3n, VO.3f (C). Scale bars,1000 μm and 200 μm. (B and D) Statistical plot of average number of protrusions per mm perimeter for the proximal and distal GB sides in DO.2n (B) and VO.3n (D). Values represent mean ± SEM (n = 5). (E and F) Gene Ontology terms of down-regulated DEGs in DO.1, DO.2n, DO.2f, VO.1 and VO.3n.

To investigate the impact of GB cells on DOs and VOs, we examined the extension of neurites from DOs and VOs within the Matrigel, which mimics the extracellular environment in our 3D models. Following co-culture, we observed a reduction in neurite density on the DO side adjacent to the GB in model 2 (Figure 3A). Quantitative analysis confirmed a significant decrease in mean neurite density between the DO distal side from GB and the proximal side from GB at day 3 after co-culturing (Figure 3B). In contrast, no significant difference in neurite density was observed in VOs in model 3 (Figures 3C and 3D). These findings suggest that GB cells adversely affect the development of brain organoids, with a more pronounced impact on DOs than VOs. After 14 days of co-culture, we conducted RNA sequencing analysis. Principal component analysis revealed a significant differential clustering between the co-culture groups and control groups (Figure S4A). Among the three models, we identified 286 upregulated genes and 130 downregulated genes in DO.1 compared to the ctrl.DO, while DO.2n exhibited the upregulation of 617 genes and downregulation of 219 genes. Similarly, DO.2f showed the upregulation of 339 genes and the downregulation of 215 genes (Figure S4). In comparison, VO.1 exhibited the upregulation of 416 genes and downregulation of 379 genes compared to the control VO group, while VO.3n showed the upregulation of 208 genes and downregulation of 326 genes. Additionally, VO.3f exhibited upregulation and downregulation of 108 genes (Figure S5). These results indicated that GB exerts a more profound influence on DOs than VOs, and the DEGs were affected by the distance of GB. In the model 1, VO.1 exhibited a significantly higher number of DEGs compared to the other two models. This phenomenon may be attributed to the co-existence of DOs, where excitatory neuronal activity in DOs promotes the migration and invasion of GB.1, ultimately leading to an augmented effect on VO.1 by GB.1.

GO enrichment analysis was performed separately for differentially downregulated genes in both DO and VO groups (Figures 3E and 3F). The enrichment results for DEGs in each group were all related to development. Notably, DO.2n exhibited a significant downregulation of genes involved in synaptic transmission, including the regulation of membrane potential, chemical synaptic transmission control, glutamate synaptic transmission, and GABAergic synaptic transmission, compared to the control group (Figure 3E). This finding suggests a dysfunction in synaptic transmission in DO.2n. Meanwhile, upregulated genes in DOs and VOs were found to be associated with extracellular matrix assembly and response to low oxygen levels (Figure S6).

**Figure 4:**
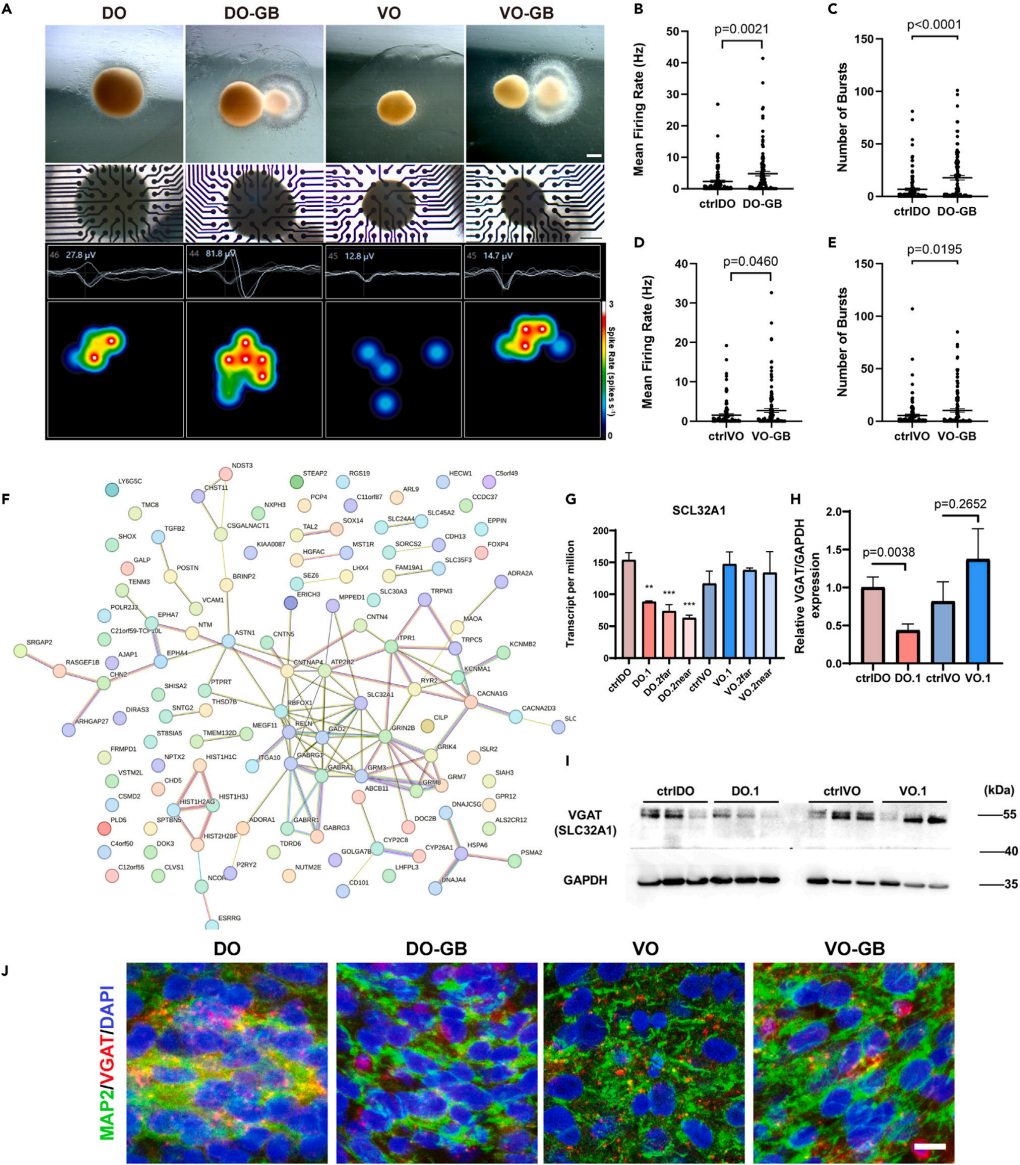
Glioblastoma cells increased neural activity in dorsal forebrain organoids (A) Morphology of organoids under stereopicroscope and phase contrast microscope view of organoids when placed on electrodes. Scale bars, 500 μm. (B and C) Statistical plot of mean firing rate and number of bursts in ctrl DO and DO-GB groups. Values represent mean ± SEM (n = 123 and 108 electrodes from N = 5 and 5 organoids). (D and E) Statistical plot of mean firing rate and number of bursts in ctrl VO and VO-GB groups. Values represent mean ± SEM (n = 127 and 112 electrodes from N = 6 and 6 organoids). (F) Protein network interaction diagram of related down-regulated DEGs in DO.2n. (G) TPM of the expression of SLC32A1 in DOs and VOs. Values represent mean ± SEM (n = 3), ∗p < 0.05, ∗∗p < 0.01, ∗∗∗p < 0.001. (H) Intensity density quantification of western blots for VGAT (SLC32A1). Values represent mean ± SEM (n = 8). (I) Immunoblotting for VGAT (SLC32A1) in ctrl.DO, DO.1, ctrl. VO and VO.1. GAPDH used as internal controls. (J) Images of DO, DO-GB, VO, and VO-GB immunostained for VGAT. Scale bars, 10 μm.

Both DO and VO exhibited an enrichment of DEGs associated with synaptic transmission, indicating that co-culturing with GB cells induces the changes in neuronal electrical activity and synaptic transmission in brain organoids (Figures 3E and 3F). Therefore, we co-cultured 14-week-old brain organoids with GB for 7 days to assess the functional implications and evaluated their spontaneous neuronal electrical activity using microelectrode arrays (MEA) recordings (Figure 4A). We recorded 123 and 108 electrodes in the ctrl DOs and DO co-cultured with GB (DO-GB), respectively. The results demonstrated that co-culture with GB led to a significant increase in firing rate in the DOs, from an average of 2.323 Hz–4.786 Hz, as well as a notable rise in the number of bursts, from 6.634 to 17.71 per electrode (Figures 4B and 4C). For VOs, we recorded 127 electrodes in the control group and 112 electrodes in the experimental group. The findings demonstrated a statistically significant but modest increase in firing rate in the VOs, from an average of 1.559 Hz–2.719 Hz, as well as the rise in the number of bursts, from 5 0.464 to 10 0.29 (Figures 4D and 4E). These findings suggest that GB cells have a stronger impact on DOs than VOs on neuronal electrical activity.

One key distinction between DOs and VOs is the ratio of excitatory/inhibitory neurons. Genes significantly downregulated in DO.2n were found to be enriched in glutamatergic and GABAergic synaptic transmission, which may contribute to the changes in electrical activity. Using protein-protein interaction networks analysis of down-regulated genes in DO.2n (Figure 4F), we identified GAD2, encoding glutamate decarboxylase2 that catalyzes GABA production, and SLC32A1 (solute carrier family 32 member 1), encoding vesicular GABA transporter, as central nodes in the network. SLC32A1 expression was robustly decrease in DO.1, DO.2f, and DO.2n after being co-cultured with GB (Figure 4G). However, no significant alterations of SLC32A1 were observed in VOs (Figure 4G), suggesting its potential role in the differential electrical activity changes between DO-GB and VO-GB. Previous studies have discovered that missense mutations in SLC32A1 lead to epilepsy and neurodevelopmental disorders through two mechanisms: altering synaptic vesicle filling and regulating synaptic short-term plasticity.^25^^,^^26^^,^^27^ Additionally, a significant reduction in the density of GABAergic neurons has been observed in the vicinity of gliomas in rats.^28^ Therefore, we detected the protein expression of SLC32A1 in DO and VO after two weeks of co-culture with GB (Figures 4H and 4I). The results showed a significant decrease in VGAT (vesicular GABA transporter) protein expression in DOs, while a slight increase was observed in VOs (Figures 4I and 4J). Collectively, our findings suggest that GB cells lead to a decrease in the expression of VGAT in DOs, which may contribute to neuronal hyper-excitability.

## Discussion

The early pathophysiology of glioblastoma is associated with seizures, which result from synaptic imbalances and hyperexcitation in neural networks.^1^ A clinical study on patients with glioblastoma revealed an intriguing finding: In patients with GBM who had no seizures at onset, an increased risk of presenting seizures during follow-up was identified in the superior frontal and inferior occipital lobe, as well as in inferoposterior regions of the temporal lobe.^29^ Meanwhile, a recently published study discovered that callosal projection neurons located in the hemisphere contralateral to primary GBM tumors promote progression and widespread infiltration.^30^ However, it remains unclear which specific types of neurons drive GBM progression due to challenges in distinguishing between excitatory and inhibitory neurons *in vivo*. To address this issue, our study established a 3D model that mimics the interaction between glioblastoma and human brain regional organoids. This model provides comprehensive insights into the characteristics of glioblastoma and their interactions with excitatory and inhibitory neurons, partially explaining epilepsy-susceptible brain regions. As our understanding of neuronal types in different brain regions improves, targeted interventions can be developed to focus on crucial points where tumors interact with neurons. This will inspire precise therapeutic strategies for various human brain regions.

Epithelial-mesenchymal transition (EMT) is a mechanism that makes GB cells invasive.^31^ During tumorigenesis, normal glial cells undergo a slight decrease in mesenchymal phenotype and potentially transition to a more mesenchymal phenotype through EMT-like processes induced by the microenvironment.^18^ Recent research has revealed that discrete glioma cells receive synaptic inputs from neurons and exhibit high invasiveness, which possess the characteristics of glutamate chemical synapses and can be significantly reduced by the antiepileptic drug AMPAR inhibitor Perampanel.^32^ Our studies suggest a potential connection between GB cells and glutamatergic synapses in Matrigel, as glioblastoma cells exhibited migratory and proliferative behavior along the axons of excitatory neurons. Neuronal activity in dorsal forebrain organoids promotes tumor invasion and infiltration, which is not observed in ventral forebrain organoids probably due to the lack of sufficient glutamatergic synapses connecting with tumor microtubules. Further studies are needed to explore the specific mechanism by which GB cells receive glutamatergic signals.

The interactions between neurons and gliomas are bidirectional, with neuronal activity promoting glioma growth while the latter augments neuronal activity.^33^ This positive feedback mechanism also triggers adjacent glioma cells, ultimately leading to the excitotoxicity and apoptosis of neurons.^34^ In our co-culture organoid models, both DOs and VOs exhibited changes in electrical activity following co-culturing with GBs. However, DOs showed particularly robust alterations. Initially, the neurites density of DOs in at proximal GB side was significantly lower than that of the distal side, possibly due to the secretory factors of GBs within the extracellular matrix. In the future, a comprehensive examination of protein interaction and signal transduction processes can be achieved by integrating single-cell sequencing, mass spectrometry and proteomics.

The current methods for quantifying GB invasion primarily involve counting the number of axons or microtubules. However, the 2D maximum projection method used to track axons in 3D is time-consuming and lacks spatial information.^35^ Additionally, measuring the distance between cell location and organoid surface can be used to quantify invasion but does not provide information on GB cell morphology. In our models, Matrigel provides an extracellular matrix for cells to migrate and proliferate outwardly, allowing the direct observation of glioma cells in vivo-like migration and invasion processes. In future studies, migration assays can be employed to remove non-migrating cells/spheres from the central region,^36^ enabling further analysis of the highly invasive cell population in glioblastoma.

In summary, by combining glioblastoma organoid, human brain organoid and 3D printing technologies, we established a 3D model to mimic the specific interaction between glioma and different types of neurons. We demonstrated the preferential crosstalk between GBs and DOs, which may lead to glioblastoma invasion and neural hyper-excitability. Therefore, these well-designed and efficient 3D models will facilitate to the discovery of new drugs for precise glioblastoma therapy in the future.

### Limitations of the study

In this project, we only used one glioma stem cell line, GSC 3264. In fact, we also used cell lines MES28 and 4062 from Jeremy N Rich’s laboratory for co-culture. However, migration and proliferation of both cell lines were restricted in Matrigel, and no significant differences were observed in axonal growth of dorsal forebrain organoids after co-culture. Therefore, the relevant data are not shown in this article. A recent study proposed that functionally connected regions differ within tumors, a difference that may be at least partially attributable to distinct glioma cell subpopulations.^37^ GSC 3264 is derived from recurrent grade 4 glioblastoma,^38^ which may partially explain the lack of related phenomena we see in other cell lines. In the future, researchers can collect large-scale diverse clinical primary glioma samples and use our model to decipher the complex molecular mechanisms underlying glioblastoma heterogeneity.

In addition, our GO analysis results revealed that GB.3 and VO.3n exhibited an enrichment of immune-related differentially expressed genes; however, this subject has not been thoroughly investigated due to the absence of an immune cell microenvironment. In future studies, brain region-specific organoids can be combined with T cells, microglia, and other immune cells to explore their effects on tumor immunity.

## STAR★Methods

### Key resources table

**Table:** 

REAGENT or RESOURCE	SOURCE	IDENTIFIER
**Antibodies**		

TBR1	Abcam	Cat# AB31940; RRID:AB_2200219
REELIN	MBL	Cat# D223-3; RRID:AB_843523
SATB2	Abcam	Cat# AB34735; RRID:AB_2301417
BRN2	GeneTex	Cat# GTX114650; RRID:AB_10619683
CTIP2	Abcam	Cat# AB18465; RRID:AB_2064130
Nestin	Abcam	Cat# AB22035; RRID:AB_446723
VGlut1	UC Davis/NIH NeuroMab Facility	Cat# 75-066; RRID:AB_2187693
GAD67	Millipore	Cat# MAB5406; RRID:AB_2278725
GFAP	Millipore	Cat# MAB360; RRID:AB_11212597
OLIG2	Millipore	Cat# AB9610; RRID:AB_570666
TUJ1	COVANCE	Cat# MMS-435P; RRID:AB_2313773
MAP2	Cell Signaling Technology	Cat# 4542S; RRID:AB_10693782
PAX6	Biolegend	Cat# 901301; RRID:AB_2749901
SOX6	Millipore	Cat# ab5805; RRID:AB_2302618
NKX2.1	Millipore	Cat# MAB5460; RRID:AB_571072
SOX2	BD Pharmingen	Cat# 561469; RRID:AB_10694256
VGAT	Synaptic Systems	Cat# 131003; RRID:AB_887869
GAPDH	Abcam	Cat# AB181602; RRID:AB_2630358
anti-Mouse IgG, 488	Invitrogen	Cat# A11001; RRID:AB_2534069
anti-Mouse IgG,594	Invitrogen	Cat# A11005; RRID:AB_2534073
anti-Rabbit IgG, 594	Invitrogen	Cat# A11012; RRID:AB_2534079
anti-Rat IgG, 488	Invitrogen	Cat# A21208; RRID:AB_2535794

**Chemicals, peptides, and recombinant proteins**		

IWP2	Sigma-Aldrich	Cat# I0536
SAG	MedChemExpress	Cat# HY-12848
FGF8	PeproTech	Cat# PHG0184
LDN193189	MedChemExpress	Cat# HY-12071
SB431542	MedChemExpress	Cat# HY-10431
FGF2	PeproTech	Cat# AF-100-18B
TGF-β	Cell Signaling Technology	Cat# 8915LF
β-mercaptoethanol	Sigma-Aldrich	Cat# M3148
EGF	PeproTech	Cat# AF-100-15-1MG
Y27632	MedChemExpress	Cat# HY-10583
B-27 supplement minus vitamin A	ThermoFisher	Cat# 12587010
B27	ThermoFisher	Cat# 17504044
GlutaMAX	ThermoFisher	Cat# 35050079
Sodium pyruvate	ThermoFisher	Cat# 11360070
GDNF	PeproTech	Cat# AF-450-10
BDNF	PeproTech	Cat# AF-450-02-10
Vitamin C	MedChemExpress	Cat# HY-B0166
Matrigel	Corning	Cat# 356230

**Critical commercial assays**		

Super ECL Detection Reagent	Yeasen	Cat# 36208ES60

**Deposited data**		

RNA-sequencing data	Genome Sequence Archive-human	HRA005089

**Recombinant DNA**		

psPAX2	Addgene	Cat# 12260
pMD2.G	Addgene	Cat# 12259
Ubic-GFP	Addgene	Cat# 28022

**Experimental models: Cell lines**		

Human embryonic stem cell line (H9)	Wicell	Agreement No. 22-W0510
Glioma stem cell	Laboratory of Jeremy N Rich	GSC 3264

**Software and algorithms**		

Graphpad Prism 8.0	Graphpad software	https://www.graphpad.com/
ImageJ 2.1.0/1.53c	ImageJ software	https://imagej.net/Contributors
R version 4.1	The R Foundation	https://www.r-project.org
STRING 12.0	STRING website	https://string-db.org/
GEPIA 2	GEPIA 2 website	http://gepia2.cancer-pku.cn/#index

### Resource availability

#### Lead contact

Further information and requests for resources should be directed to and will be fulfilled by the Lead Contact, Zhicheng Shao (zcshao@fudan.edu.cn).

#### Materials availability

This study did not generate any unique new reagent. All reagents used in this study are commercially available.

#### Data and code availability

• RNA-seq data of this study have been deposited at Genome Sequence Archive and are publicly available [GSA]: [HRA005089] (https://bigd.big.ac.cn/gsa-human/browse/HRA005089). Accession numbers are listed in the key resources table.
• This paper does not report original code.
• Any additional information required to reanalyze the data reported in this paper is available from the lead contact upon request.

### Experimental model and study participant details

#### Cell lines

Human embryonic stem cell line (H9) was obtained from WiCell company (Agreement No. 22-W0510) and cultured in Nutristem medium. GSC 3264 (patient age and sex: 65 years, female; tumor grade and transcriptional subtype: recurrent glioblastoma grade IV, classical) was obtained from the laboratory of Jeremy N Rich^38^ and cultured as neurosphere in the Neurobasal medium supplemented with 2% B27 supplement without vitamin A, 1% L-glutamine, 1% sodium pyruvate, 20 ng/mL FGF2 and 20 ng/mL EGF. Cell lines were cultured in 37°C and 5% CO_2_ conditions.

### Method details

#### Preparation of 3D printed mold

The model of the mold was drawn by CAD and printed using a highly rigid PLA(Polymarker). The g.code format file was generated by slice processing. Start the 3D printer (RAISE 3D) and check the set parameters before printing. The UV-curing optical gel NOA68(Norland) is suitable for bonding 3D molds to Petri dishes. The bottom surface of the 3D mold was coated with NOA68, cured by UV light irradiation, and then sterilized with 75% alcohol immersion and checked for complete adhesion. The alcohol was removed prior to use and after UV irradiation in a biosafety cabinet, the organoids could be co-cultured in the mold.

#### Culturing and maintenance of human ESCs

The H9 human embryonic stem cell line obtained from WiCell was maintained in Nutristem medium (Biological Industries, 05-100-1A) on 1% Matrigel (Corning, 354230) pre-coated plates and passaged when colonies reached about 90% confluency using TrypLE (Gibco, 12604021) at the ratio 1:5. Cells were checked for normal karyotype and were mycoplasma-free. Unless otherwise specified, organoids shown were generated from H9 cells below passage 60. All cultures were maintained in 5% CO_2_ incubators at 37°C.

#### Generation of dorsal/ventral forebrain organoids

To generate dorsal forebrain organoids, H9 hESCs were exposed to DMEM medium including 20% knockout serum replacement (Gibco, 12604021), 1%Glutamax (Gibco, 35050061), 1% penicillin-streptomycin (Gibco, 15140122), 10 μM β-mercaptoethanol (SigmaAldrich, M3148), 10 μM SB431542 (MCE, HY-10431), and 0.1 μM LDN193189 (Sigma-Aldrich, SML0559) after dissociated into single cells. 96-well U-bottom low-attachment plate (Corning, CLS7007) was used to generate organoids of uniformly size, with an initial cell count of 10,000 per well. Additionally, the medium was supplemented with 10 μM Rock inhibitor Y-27632 (Selleckchem, S1049) for the first two days. Organoids were then transferred to six-well plates, each well containing 4–6 brain organoids. The six-well plates were placed on an orbital shaker and cultured in suspension at 75 rpm. On the 7th day, spheroids were transferred to Neurobasal medium (Gibco, 21103049) supplemented with 1× Glutamax, 1× penicillin-streptomycin, 1× NEAA (Gibco, 11140050), 2% B27 (Gibco, 17504044), 20 ng/mL bFGF (Peprotech, AF-100-18B) and 20 ng/mL EGF (R&D Systems, 236-EG). On day 21, the medium was altered into Neurobasal medium supplemented with 1× Glutamax, 1× penicillin-streptomycin, 1× NEAA, 2% B27, 10 ng/mL brain-derived neurotrophic factor (BDNF, Peprotech, 450-02), 10 ng/mL glial cell line-derived neurotrophic factor (GDNF, Peprotech, AF-450-10) and 200 μM L-Ascorbic Acid (Sigma-Aldrich, 508 A8960). The medium changed every other day for long-term maintenance.

For ventral forebrain organoids, the first week of culture medium was based on the culture medium of the first week of dorsal forebrain organoids, with the addition ventralization-inducing molecules of 0.1 μM SAG (Sigma, SML1314) and 5 μM IWP2(Sigma, I0536).The second week is DMEM medium including 20% knockout serum replacement, 1%Glutamax, 1% penicillin-streptomycin, 10 μM β-mercaptoethanol, 0.1 μM SAG and 5 μM IWP2. The third week is Neurobasal medium supplemented with 1× Glutamax, 1× penicillin-streptomycin, 1× NEAA, 2% B27, 0.1 μM SAG and 20 ng/mL FGF8(PeproTech, PHG0184). The medium for long-term maintenance culture after the fourth week is the same as dorsal forebrain organoids.

#### Maintenance of patient derived GSCs of and generation glioblastoma organoids

Glioma stem cells line 3264 was obtained from the laboratory of Jeremy N Rich and cultured as sphere in the Neurobasal medium supplemented with 2% B-27 supplement minus vitamin A (Gibco,12587010), 1% GlutaMAX (Gibco, 35050061), 1% sodium pyruvate (Gibco, 11360070), 20 ng/mL FGF2 and 20 ng/mL EGF. The spheroids formed by glioma cells were cultured in six-well plates. When the spheroid size becomes larger, the growth of glioma stem cell lines slows down and requires passage. Use Tryple to dissociate the spheroids into small cell clumps and continue culturing. Unpassaged spheroids were picked and transferred to 48-well plates for further culture to mimic the heterogeneity of gliomas. The spheroids were then cultured as organoids embed in Matrigel to observe their migration and spreading behavior.

#### Co-culture of dorsal/ventral forebrain and glioblastoma organoids in 3D printed mold

The prepared molds were sterilized by UV irradiation for 30 min, the Pipette tips were cut to transfer the organoids for co-culture. The 3-5mm spacing was reserved between organoids and the grooves were filled with a gentle drop of liquid Matrigel (about 400μL) to ensure complete submersion of each organoid. The position of the organoids was adjusted so that the centers of the organoids were on the same plane for easy subsequent observation of the cell phenotype. The medium used for co-culture was brain organoid maturation stage medium. The duration of co-culture was determined according to cell migration interaction distance and state, usually 12–16 days, and the co-culture days for RNA-sequencing organoids are all two weeks.

#### Lentivirus production and transduction

Reporter tags such as ubic-GFP was expressed in the cytoplasm of GSCs using lentiviral transduction. In short, target vectors and packaging plasmids (pMD2.G, and psPAX2) were transfected into HEK293TS cells using calcium chloride and HEPES. After 8 h, the medium was changed, and the virus was collected after another 8 h. The freshly harvested virus was used to transduce target cells in a 1:1 ratio for 72–96 h.

#### Immunostaining

Organoids were fixed with 4% paraformaldehyde for 30 min. Fixed organoids were then washed in PBS for 3 times and dehydrated in 30% sucrose overnight at 4°C. Dehydrated organoids were added in optimal cutting temperature (OCT) compound and frozen for the sectioning at −20°C. Then they were cut into 20 μm thick sections. Cryosections were washed with PBS to remove excess OCT compound and blocked blocking solution (3% BSA, 0.3% Triton X-100 diluted in PBS) for 1 h at room temperature. The sections were then incubated overnight at 4°C with primary antibodies diluted in blocking solution. The primary antibodies include used for immunostaining TBR1(Rb,1:1000), REELIN(Mouse,1:300), SATB2(Rb,1:500), BRN2(Rb,1:1000), CTIP2(Rat,1:500), CUX1(Rb,1:200), VGlut1(Mouse,1:250), GAD67(Mouse,1:50), GFAP(Mouse,1:500), OLIG2(Rb,1:200), TUJ1(Mouse,1:1000), MAP2(Rb,1:1000), PAX6(Rb,1:300), SOX6(Rb,1:1000), NKX2.1(Mouse,1:1000) and SOX2(Mouse,1:50). After 3 times washing with PBS, sections were incubated in corresponding secondary antibodies including anti-Mouse IgG, 488(Gt,1:1000), anti-Mouse IgG,594(Gt,1:1000), anti-Rabbit IgG, 594(Gt,1:1000), anti-Rat IgG, 488(Donkey,1:1000) for 1 h and then washed with PBS three times at room temperature. Finally, sections were sealed with VECTASHIELD (H-1000, VECTOR) and Olympus FV3000 was used for imaging.

#### RNA-seq analysis

For RNA sequencing, organoids were cultured in triplicates for each condition and total RNA extraction was performed using RNAiso Plus (Takara, 9109) according to the manufacturer’s protocol. Agilent 2100 Bioanalyzer and Agilent RNA 6000 Nano Kit were used for concentration and integrity detection. Purity was measured using the Nano Drop 2000&8000 spectrophotometer. The transcriptome sequencing was conducted by Annoroad Co., Ltd (Shanghai, China). The libraries were sequenced on an Illumina NovaSeq 6000 system and 150-bp paired-end reads were generated. Raw data (raw reads) of the fastq format were first processed using Trimmomatic version 0.36, and about 40–65 million (M) raw reads for each sample were generated. Then about 40–65 M clean reads for each sample remained, which were obtained for downstream analyses by removing reads containing adapter, reads containing ploy-N and low-quality reads from raw data. The clean reads were mapped to the human genome using HISAT2 version 0.36. The transcripts per million (TPM) values of each gene was calculated and the read counts of each gene were obtained by featureCounts. Differentially Expressed Genes (DGE) was determined using the DESeq2. In DO and VO groups, log-fold-change(logFC) > 1 and false discovery rate (FDR) < 0.05 were considered statistically significant, while in GB group, logFC >2 were set as the threshold. Further, to investigate the potential functional differences that DEGs can infer, we performed gene ontology analysis using clusterProfiler package to identify the GO terms associated with DEGs previously determined. After calculating the fold change value of gene expression difference between group, Gene set enrichment analysis (GSEA) was performed to obtain functional pathways in MsigDB databases that significantly associated with characteristics of different groups, with p value <0.05 and FDR <0.25. Previously identified DEGs were used to build Protein-Protein Interaction (PPI) network based on STRING database.

#### Western blotting

To detect glioblastoma-induced changes in the forebrain organoids, protein samples of DOs and VOs were collected after 14 days co-culture with GBs. Organoids were washed three times with 1 × PBS and lysed with 500 μL of RIPA buffer (Sigma-Aldrich) with protease inhibitor (Sigma-Aldrich) and phosphatase inhibitor (Sigma-Aldrich). After centrifuged for 10 min at 12,000 rpm, protein contents in the supernatant were quantified using Bio-Rad Protein Assay Dye Reagent Concentrate (Bio-Rad). Protein samples were prepared by mixing 5 × SDS buffer (250 mM Tris·HCl, pH 6.8, 10% SDS, 0.2% bromophenoblue, 50% glycerol, 4% 2-mercaptoethanol) with 30 μg of cell lysates and separated using SDS-PAGE 8% gels. After completion of electrophoresis, proteins were transferred onto PVDF membrane (Millipore). Membranes were blocked by 3% BSA in TBS-T (50 mM Tris·HCl, pH 7.4, 150 mM NaCl, and 0.05% Tween 20) for 1 h and then incubated with primary antibody antibodies VGAT (rabbit, 1:500, Synaptic Systems) and GAPDH (rabbit, 1:5,000, Abcam, ab181602) overnight at 4°C. The membranes were incubated in secondary antibodies (1: 2000) for 1h at room temperature. Then Chemiluminescence (Yeasen, Super ECL Detection Reagent, 36208ES60) was used to detect the signals. Finally, intensities of the bands of the correct molecular weights were quantified and were first normalized to that of GAPDH and then calculated to obtain the ratio of total levels of VGAT in different organoids.

#### Microelectrode arrays (MEA) recording

MEA was used to detect the neuronal firing activity. Each well of six-well MEA plates (Axion Biosystems) contains 64 low-impedance platinum microelectrodes (0.04 MΩ micro/electrode) with a diameter of 30 mm, which are spaced 200 mm apart. The plate was pre-coated with Matrigel 1:100 diluted in the DMEM medium at an incubator for 30 min. Organoids co-cultured with GB for 2 weeks were then placed onto the plate to cover the microelectrodes and the fresh medium was added. Recordings for 10–15 min were performed using the default neural activity settings from a Maestro pro MEA system and AxIS Software Spontaneous Neural Configuration (Axion Integrated Studio Navigator 1.5, Axion Biosystems). Mean firing rate was measured by counting spikes from active electrodes in each well per minute. Spike and burst detection were further analyzed and plotted by Neural Metric Tool version 2.2.3.

### Quantification and statistical analysis

Data are presented as mean ± SEM. The unpaired two-tailed t-test using GraphPad Prism software version 7.0 were used to determine the statistical significance. The significance levels are ∗p < 0.05, ∗∗p < 0.01 and ∗∗∗p < 0.001. All the statistical tests and biological replicates are explained in the figure legends.
