# Supplementary material for: Modeling the precise interaction of glioblastoma with human brain region-specific organoids
Source: iScience. 2024 Feb 5;27(3):109111. doi: 10.1016/j.isci.2024.109111 (PMC10882168; doi:10.1016/j.isci.2024.109111)
Supplement: Document S1. Figures S1–S6 [file mmc1.pdf]

iScience, Volume *27*

Supplemental information

Modeling the precise interaction of glioblastoma with human brain region-specific organoids

Qi Fan, Hanze Wang, Tianyi Gu, Huihui Liu, Peng Deng, Bo Li, Hui Yang, Ying Mao, and Zhicheng Shao

Figure S1

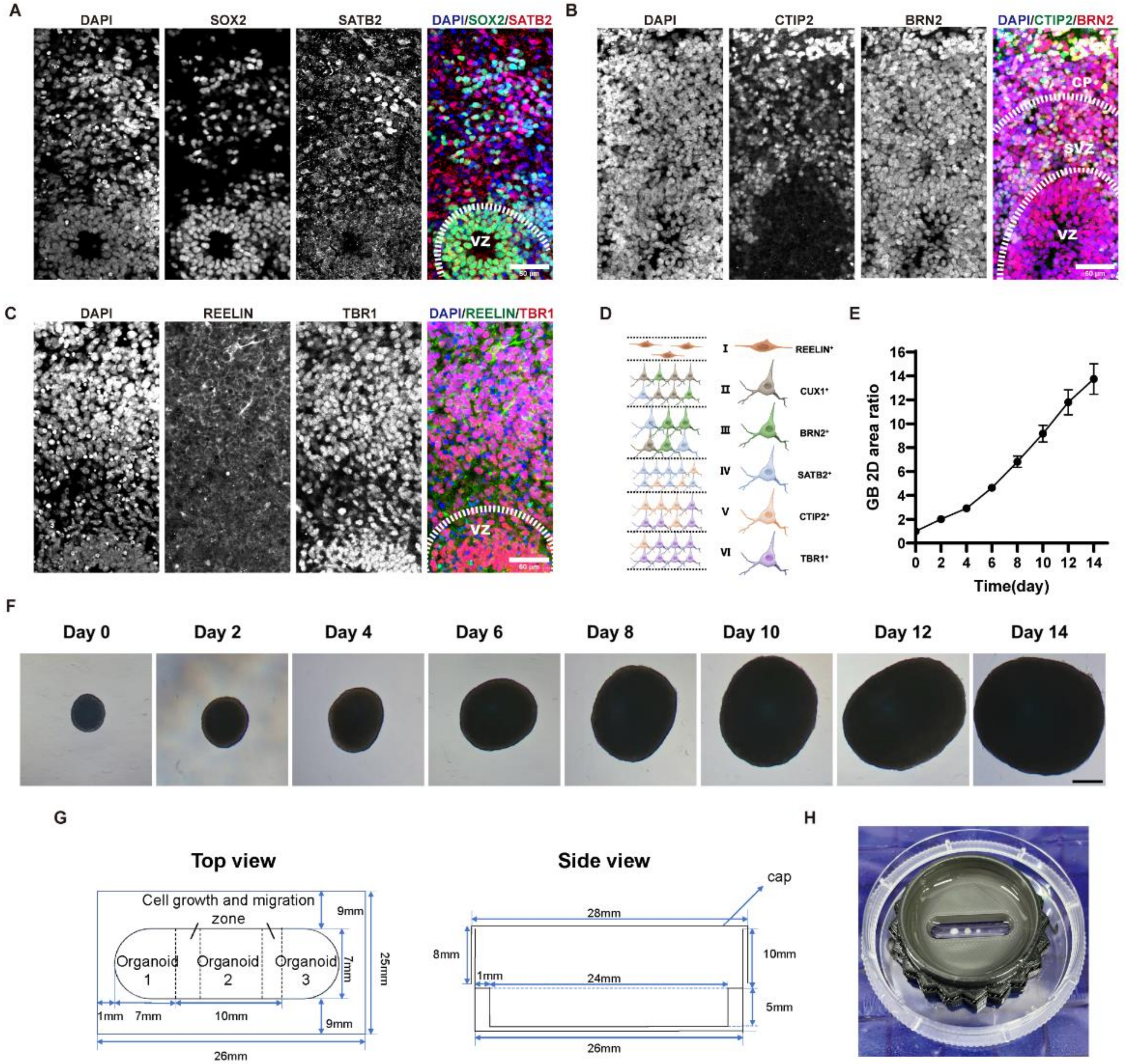

Figure S1. Establishment of dorsal/ventral forebrain organoids, glioblastoma organoids, and 3D-printed mold, Related to Figure 1. (A-C) Representative immunostained images of the cortical layer markers in DO. Scale bars, 50 μm. (D) Schematic representation of the neuronal markers in the six cortical layers (I–VI). (E) Quantification of the ratio of the measured 2D area at each time point to the 2D area at time point 0 of the same GB. Values represent mean ± SEM (n = 7). (F) Brightfield images of individual GB during a 2-week period. Scale bar, 100 μm. (G) Top and side views of 3D printing mold design dimensions. (H) Image of organoids co-cultured within molds.

Figure S2

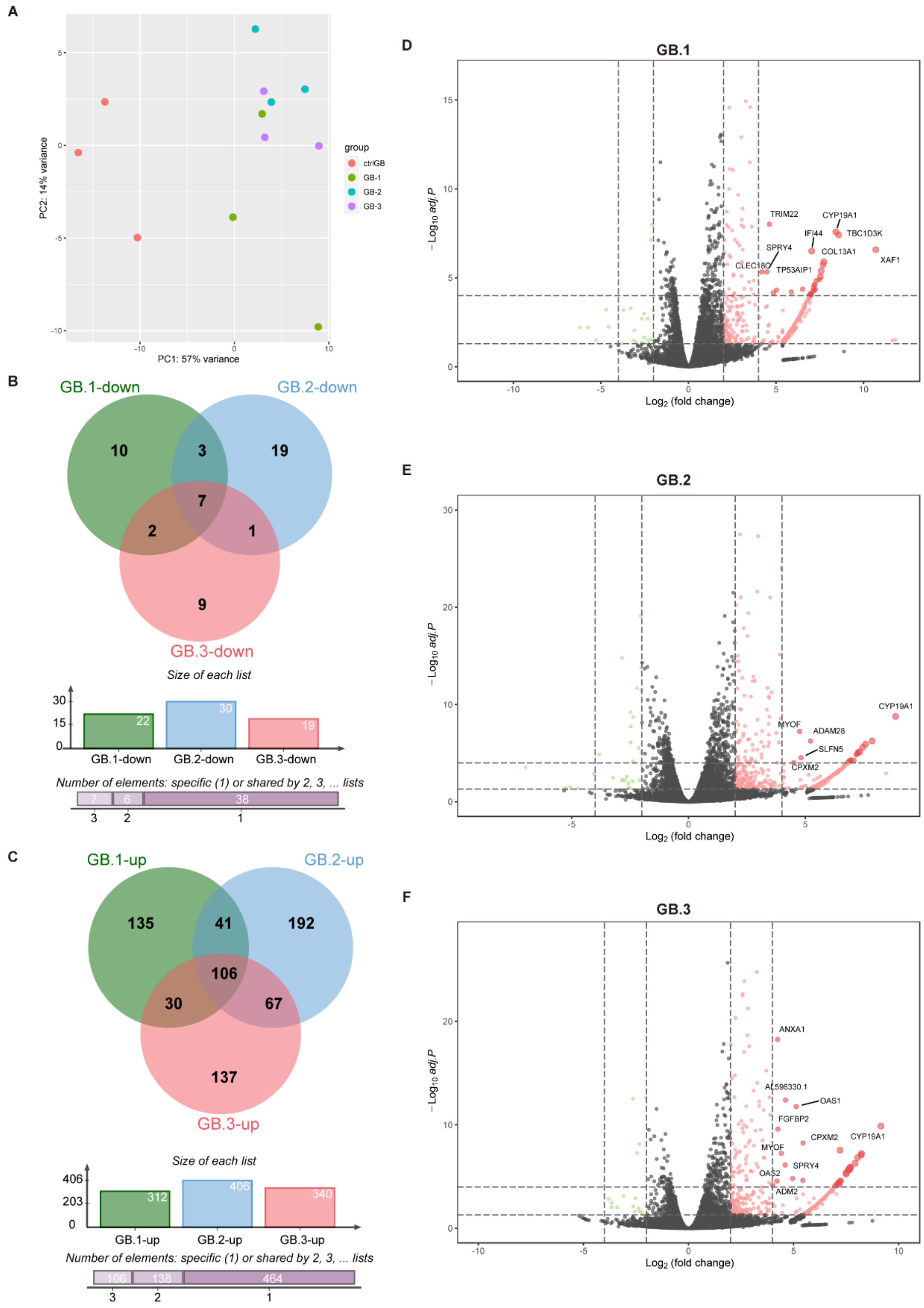

Figure S2. RNA-Seq data analyses of GB group, related to Figure 2. (A) PCA of GB.1, GB.2, GB.3 vs ctrl.GB. (B) Venn diagram of down-regulated DEGs in GB groups. (C) Venn diagram of upregulated DEGs in GB groups. (D-F) Volcano plots comparing GB.1, GB.2, GB.3 vs ctrl.GB.

Figure S3

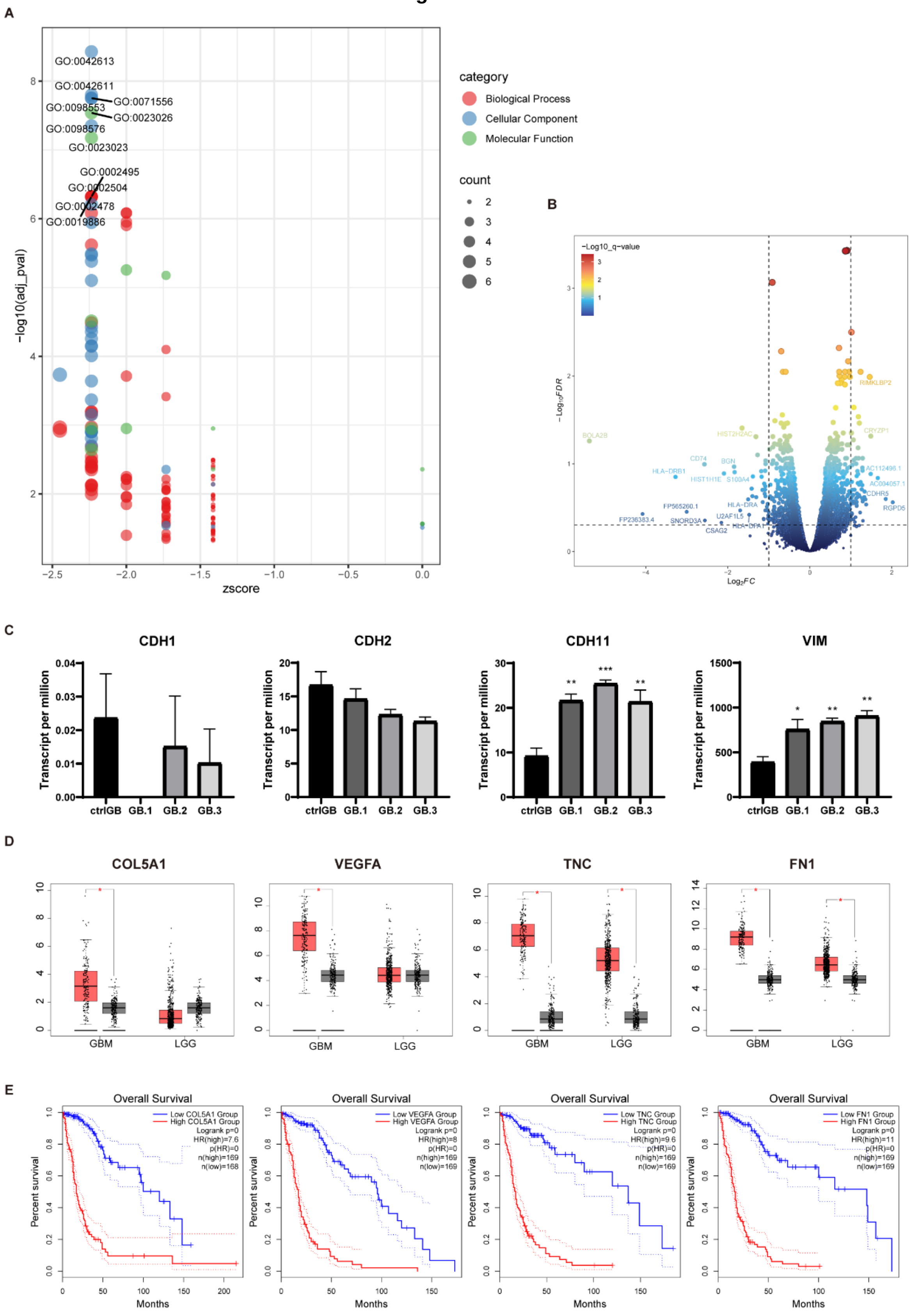
A
B
C
D
E
category
Biological Process
Cellular Component
Molecular Function
count
zscore
−log10(adj_pval)
GO:0042613
GO:0042611
GO:0071556
GO:0098553
GO:0023026
GO:0098576
GO:0023023
GO:0002495
GO:0002504
GO:0002478
GO:0019886
−Log10_q−value
RIMKLBP2
CRYZP1
BOLA2B
HIST2H2AC
CD74
BGN
HLA−DRB1
HIST1H1E
S100A4
AC112496.1
AC004057.1
CDHR5
RGPD5
FP565260.1
HLA−DRA
FP236383.4
SNORD3A
U2AF1L5
CSAG2
HLA−DPA1
CDH1
CDH2
CDH11
VIM
Transcript per million
ctrlGB
GB.1
GB.2
GB.3
COL5A1
VEGFA
TNC
FN1
GBM
LGG
Overall Survival
Low COL5A1 Group
High COL5A1 Group
Logrank p=0
HR(high)=7.6
p(HR)=0
n(high)=169
n(low)=168
Low VEGFA Group
High VEGFA Group
Logrank p=0
HR(high)=8
p(HR)=0
n(high)=169
n(low)=169
Low TNC Group
High TNC Group
Logrank p=0
HR(high)=9.6
p(HR)=0
n(high)=169
n(low)=169
Low FN1 Group
High FN1 Group
Logrank p=0
HR(high)=11
p(HR)=0
n(high)=169
n(low)=169
Percent survival
Months

Figure S3. RNA-Seq data analyses of GB.2 vs GB.3 and genes implicated in glioblastoma in both patients and our model, related to Figure 2.
(A) Gene Ontology terms of DEGs in GB.2 vs GB.3.
(B) Volcano plots comparing GB.2 vs GB.3.
(C) TPM of the expression of CDH1, CDH2, CDH11 and VIM in GBs. Values represent mean ± SEM (n = 3). $*p < 0.05$, $**p < 0.01$, $***p < 0.001$.
(D) Expression of COL5A1, VEGFA, TNC and FN1 in tumor tissues and normal tissues of GBM (num(T)=163; num(N)=207) and LGG (num(T)=518; num(N)=207) patients, ordinate is log2 (TPM + 1). Source: GEPIA2.
(E) Survival curves for GBM patients, group cutoff: quartile. Source: datasets GBM and LGG, GEPIA2.

Figure S4

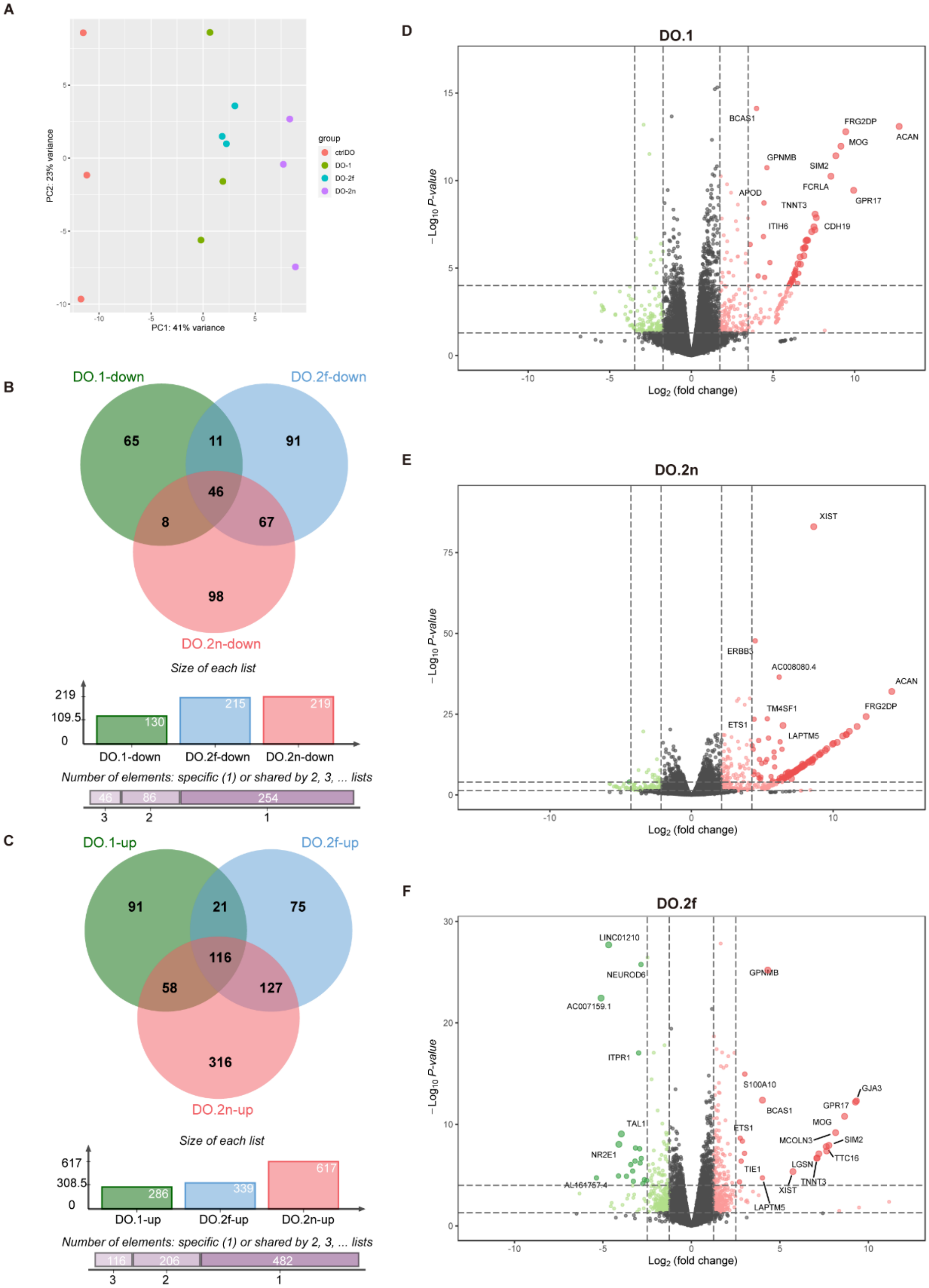

Figure S4. RNA-Seq data analyses of DO group, related to Figure 3. (A) PCA of DO.1, DO.2n, DO.2f and ctrl.DO. (B) Venn diagram of down-regulated DEGs in DO groups. (C) Venn diagram of upregulated DEGs in DO groups. (D-F) Volcano plots comparing DO.1, DO.2n, DO.2f vs ctrl.DO.

Figure S5

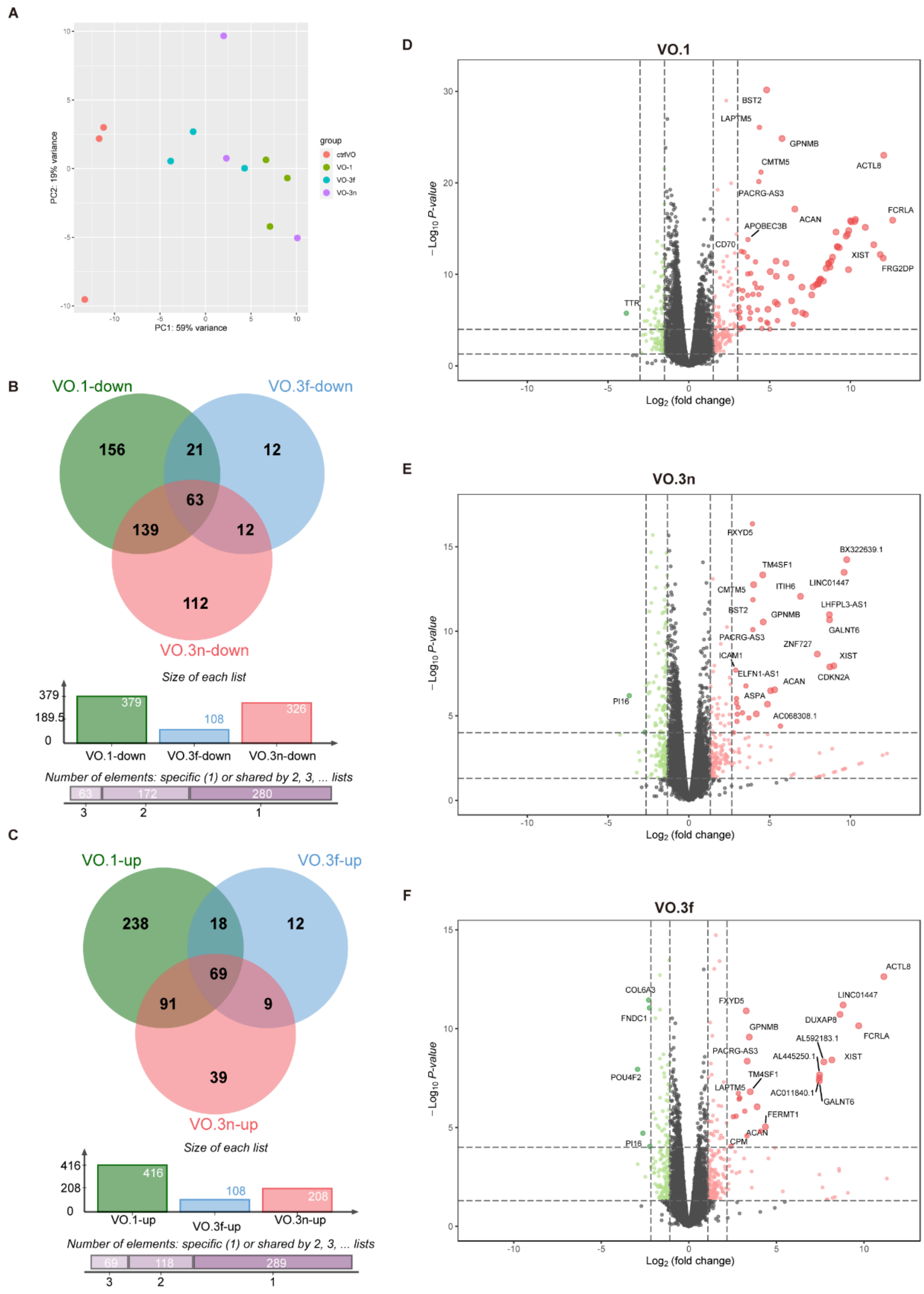

Figure S5. RNA-Seq data analyses of VO group, related to Figure 3. (A) PCA of VO.1, VO.3n, VO.3f and ctrl.VO. (B) Venn diagram of down-regulated DEGs in VO groups. (C) Venn diagram of upregulated DEGs in VO groups. (D-F) Volcano plots comparing VO.1, VO.3n, VO.3f vs ctrl.VO.

Figure S6

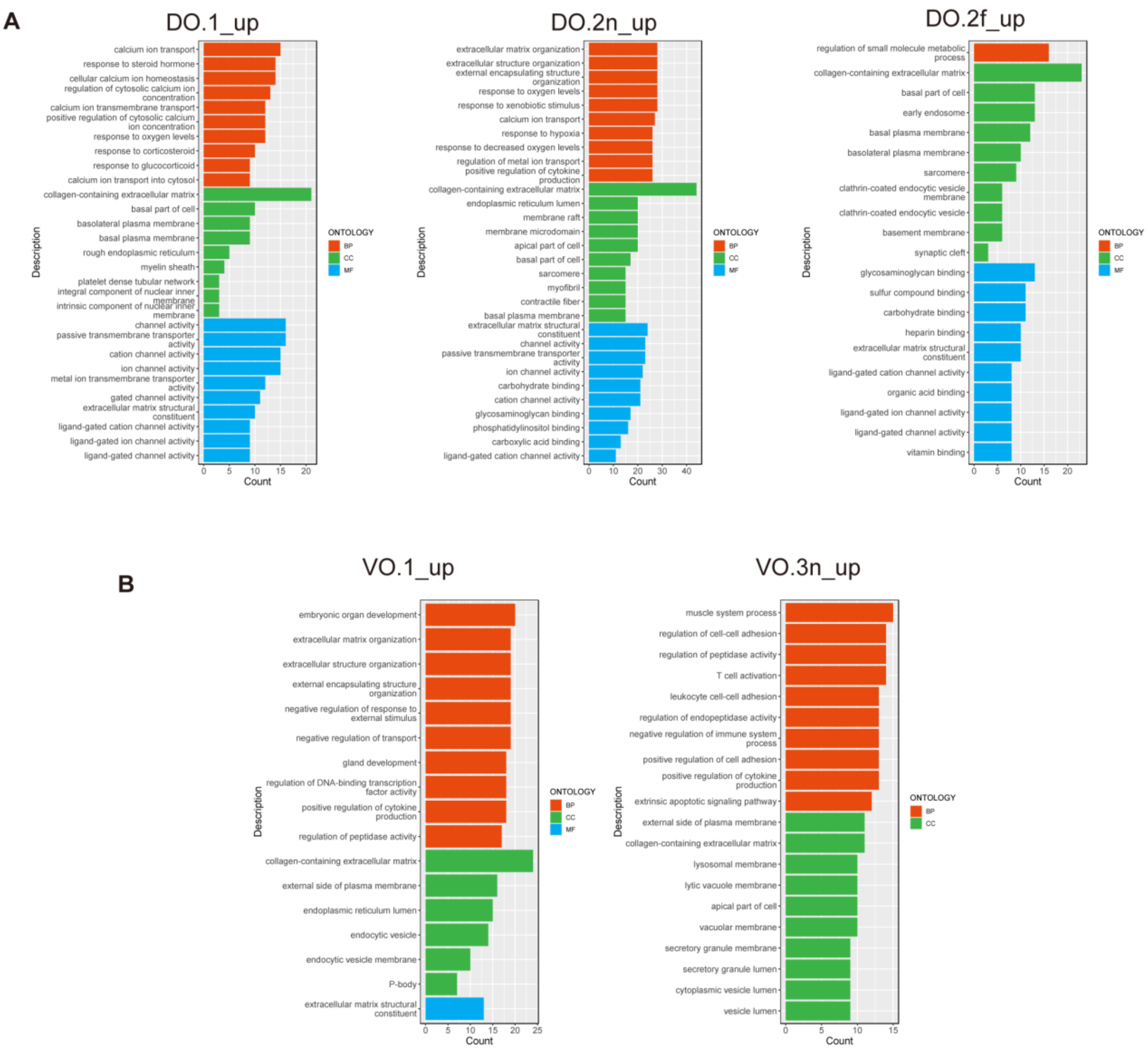

Figure S6. Gene oncology enrichment analysis of upregulated DEGs in DO and VO groups, related to Figure 3. (A) Gene Ontology terms of up-regulated DEGs in DO.1, DO.2n and DO.2f. (B) Gene Ontology terms of up-regulated DEGs in VO.1 and VO.3n.
